# Conservation of the Nucleotide Excision Repair Pathway: Characterization of Hydra *Xeroderma Pigmentosum Group F* Homolog

**DOI:** 10.1371/journal.pone.0061062

**Published:** 2013-04-08

**Authors:** Apurva Barve, Saroj Ghaskadbi, Surendra Ghaskadbi

**Affiliations:** 1 Division of Animal Sciences, Agharkar Research Institute, Pune, India; 2 Department of Zoology, University of Pune, Ganeshkhind, Pune, India; St. Georges University of London, United Kingdom

## Abstract

Hydra, one of the earliest metazoans with tissue grade organization and nervous system, is an animal with a remarkable regeneration capacity and shows no signs of organismal aging. We have for the first time identified genes of the nucleotide excision repair (NER) pathway from hydra. Here we report cloning and characterization of hydra homolog of *xeroderma pigmentosum group F (XPF)* gene that encodes a structure-specific 5′ endonuclease which is a crucial component of NER. *In silico* analysis shows that hydra XPF amino acid sequence is very similar to its counterparts from other animals, especially vertebrates, and shows all features essential for its function. By *in situ* hybridization, we show that hydra *XPF* is expressed prominently in the multipotent stem cell niche in the central region of the body column. Ectoderm of the diploblastic hydra was shown to express higher levels of *XPF* as compared to the endoderm by semi-quantitative RT-PCR. Semi-quantitative RT-PCR analysis also demonstrated that interstitial cells, a multipotent and rapidly cycling stem cell lineage of hydra, express higher levels of *XPF* mRNA than other cell types. Our data show that *XPF* and by extension, the NER pathway is highly conserved during evolution. The prominent expression of an NER gene in interstitial cells may have implications for the lack of senescence in hydra.

## Introduction

Hydra, a fresh water hydrozoan, is a well-known example of phylum Cnidaria, the earliest animal phylum with true tissue grade organization and nervous system [1]. It is diploblastic with a simple cylindrical body bearing a conical hypostome with tentacles at one end and a flattened, mucus-secreting basal disc at the other [2]. It has a remarkably high capacity of regeneration and a propensity to continuously asexually propagate by budding. Hydra has three stem cell lineages: ectodermal and endodermal epithelial stem cells and interstitial stem cells [3]. The stem cells divide continuously and excess cells thus generated are pushed into developing buds or sloughed off from either end of the body so that the overall size of the animal remains constant [3]. An individual hydra polyp displays no sign of senescence and has been shown to survive for over four years without any decrease in physiological activities or rate of asexual and sexual reproduction [4]. Many developmental mechanisms, signalling pathways and genes that are present in hydra are conserved up to higher phyla [5]. While various aspects of hydra biology, including axis determination, patterning, and cell signalling are currently being investigated [5], nothing is known about DNA repair in this animal. Though ultraviolet (UV) radiation-induced DNA damage and its repair has been reported in the branching coral *Stylophora* [6], the pathways or proteins employed for DNA repair in Cnidaria in general or hydra in particular are not well studied. Here we report identification, isolation, cloning and partial characterization of *XPF*, an endonuclease of the NER pathway, from hydra.

DNA in cells is constantly subjected to assaults from internal and external agents [7]. Organisms have evolved mechanisms to overcome various types of DNA damage. Among these, NER is a versatile pathway for repair of helix distorting lesions such as those caused by UV or some chemotherapeutic drugs [8]. This pathway is present across all organisms from bacteria to humans. In prokaryotes, NER is carried out by the UvrABC system [9] while combinations of elements from either or both prokaryotic and eukaryotic NER are seen in various members of archaea [10]. Among eukaryotes, NER is well studied in yeast and mammals [11]. XP group of proteins are the principal orchestrators of eukaryotic NER. The name XP is eponymous with the genetic disorder where patients are highly prone to skin cancers, extremely sensitive to sun-exposure and have rough, pigmented skin. NER involves several sequential steps like recognition of damage, recruitment of relevant proteins to the site, localized DNA unwinding, dual incision of DNA strand on either side of damage and removal of the resulting fragment followed by gap filling and ligation [12]. While presence of one of the NER genes, *XP group B*, has been reported from the sponge *Geodia* [13], the pathway remains largely uncharacterized in early-evolved animal phyla including Cnidaria. *XPF* encodes the endonuclease that cuts the damaged DNA strand about 22–25 bases on 5′ side of the lesion during NER [14]. XPF forms a functional heterodimer with the protein Excision Repair Cross Complementing protein (ERCC) 1. The XPF-ERCC1 complex is involved in several processes other than NER, like interstrand cross-link repair [15], double strand break repair [16], transcription of certain groups of genes, removal of 5-methyl cytosine and chromatin remodelling [17], [18]. Thus, certain mutations of *XPF* can affect several physiological processes including the somatotroph axis and can lead to a segmental progeroid syndrome where patients suffer from spontaneous accelerated aging [19].

Here we show that a homolog of *XPF* is present in hydra and possesses various conserved features of XPF proteins. It clusters with its homologs from other animals in phylogenetic analysis and is expressed predominantly in the multipotent stem cells of hydra.

## Materials and Methods

### Hydra Culture

*H. vulgaris* Ind-Pune, *H. magnipapillata* and *H. magnipapillata* sf-1 strain animals were cultured as described previously [20].

### Identification, Isolation and Cloning of *XPF* from *H. vulgaris* Ind-Pune

The *H. vulgaris* AEP EST database was screened to find sequences matching with *XPF.* Two ESTs were identified from the screen. Complete CDS of hydra *XPF* was obtained by overlapping of the two ESTs; amplified from cDNA of *H. vulgaris* Ind-Pune (Fw: ATGTCAGGTGCATTTGATACATTGTTAAAG, Rev: TCACTTGCTTGTTTTTTTAATACTAGTTG); cloned in pGEM-T (Promega, USA) and sequenced.

### Sequence Analysis of Hydra *XPF*

The degree of similarity between *XPF* from hydra and other species was analysed by BLAST (http://blast.ncbi.nlm.nih.gov). CDS was translated *in silico* and the protein sequence was screened to predict the presence of sub-cellular localization signals using the PSORT-II package (http://psort.hgc.jp/form2.html). SMART (http://smart.embl-heidelberg.de/) was used to find conserved domains in the putative protein. Swiss Model (http://swissmodel.expasy.org) [21] was used for homology modelling of hydra XPF protein structure. The Swiss PDB Viewer software Deep View was used for comparing hydra XPF models with solved structures from the protein data bank (PDB) and calculating root mean square deviation (RMSD) value of superimposed peptides. Phylogenetic analysis of XPF proteins was carried out using following sequences: *Trypanosoma cruzi* CL Brener (XP_808683), *Trichoplax adhaerens* (XP_002114393), *H. vulgaris* Ind-Pune (ADQ08682.1), *H. magnipapillata* (XP_002161035), *Nematostella vectensis* (XP_001631128), *Caenorhabditis elegans* (NP_496498), *Drosophila melanogaster* (NP_525068), *Strongylocentrotus purpuratus* (derived from XR_026220.1/XR_025965.1), *Saccoglossus kowalevskii* (XP_002731700), *Danio rerio* (NP_956079), *Xenopus laevis* (NP_001086576), *Mus musculus* (NP_056584), *Homo sapiens* (NP_005227.1) and *Arabidopsis thaliana* (NP_198931). The sequences were aligned using MUSCLE program in MEGA5.05 [22] and phylogenetic trees (bootstrap = 1000) based on Neighbour Joining (NJ) and Maximum Parsimony (MP) methods were constructed in MEGA while Maximum Likelihood (ML) tree was constructed with PhyML [23].

### *In situ* Hybridization

*In situ* hybridization using biotin labelled riboprobes was carried out for localization of *XPF* mRNA in whole, non-budding hydra as described previously [24] but with slight modifications. Biotin labelled sense and anti-sense riboprobes were generated as per manufacturer’s instructions (Roche, Germany) for 350 bp region of hydra *XPF* CDS (Fw: TGGTCGATTATATTCACAGTGTTT; Rev: GGTTCCGTTTGATCTGCACT). Hybridisation was carried out at 60°C for 16–36 hrs, the samples were washed to remove excess probe and incubated overnight with AP-tagged streptavidin. After thorough washing the signal was detected using NBT-BCIP as substrate and reaction was stopped simultaneously for both sense and anti-sense samples.

### Tissue Manipulation and Treatments

Ectoderm and endoderm of hydra were separated using procaine at low pH as previously described [25]. sf-1 hydra devoid of interstitial cells were generated by maintaining about 50 animals at 28°C for 8 days. An equal number of sf-1 hydra were kept at 18°C as controls. At the end of the treatment, 2–3 hydra from each set were macerated using about 50 µl of 1∶1:13 acetic acid: glycerol: water solution. Cytological examination of both macerated samples was carried out to check for the presence of interstitial cells. Two groups of wild-type *H. magnipapillata* subjected to the same treatments as sf-1 samples served as controls for effects of high temperature on hydra.

### Semi-quantitative Reverse Transcriptase PCR (RT-PCR)

RNA extraction from ectodermal and endodermal layers and heat-treated hydra; cDNA synthesis and RT-PCR were carried out following standard protocol [25]. For all RT-PCR experiments, cDNA samples were equilibrated using the housekeeping gene actin (Fw: GTTGACAATGGCTCCGGTAT; Rev: CATCGTACTCCTGCTTGCTG). *nb042* whose expression is restricted to the differentiating nematocytes was used as an ectodermal marker and *Hydkk1/2/4-C* which is expressed only in the gland cells was used as endodermal marker to verify purity of respective separated tissue samples [25]. cDNA derived from the three cell types of *H. vulgaris* AEP was a kind gift from Anna-Marie Böhm and Georg Hemmrich; Kiel, Germany. To ascertain purity of samples of each cell-type, PCRs were carried out for *ks1* (Fw: GTATTACATTCAAGATTCAGAG; Rev: GTTTGCTTCTTTTAACTTTAAGG); *TRG82* (Fw: TAGCATTTATTCCTAAATATGAAAAG; Rev: TCTGCAGTTTGGTATTGTACG); *TRG111* (Fw: TGTCATGAAAGTAATGGAAG; Rev: GGCACTTCTTTTATTTCTTCTC) which are marker genes for ectodermal epithelial cells, endodermal epithelial cells and interstitial cells, respectively. *Cnnos1* (Fw: GACTTATAAGACTGATACTACC; Rev: CATGTGTAGGCTCTAAGTATTG), the nanos homolog of hydra expressed only in multipotent interstitial stem cells and germ-line cells [26] was used as a marker for interstitial cells. Primers corresponding to the 350 bp region of hydra *XPF* mentioned above were used for *XPF* RT-PCR. Band intensities of *XPF* were normalized using band intensities of actin. In case of RT-PCR for the three cell types, cDNA obtained from just one round of sorting was used and the PCR was carried out twice using the same cDNA. For all other experiments, PCRs for each set of samples were repeated at least three times and the experiment to generate the respective samples was carried out in triplicate. Standard deviation was calculated using the average normalized intensities for each set.

## Results

### Hydra *XPF* is Very Similar to its Vertebrate Counterparts and the Predicted Protein Possesses Nuclear Localization Signals (NLS), ERCC4 Domain and Nuclease Motif

**Figure 1:**
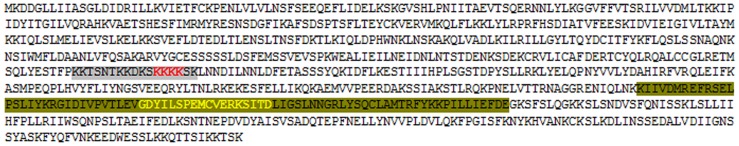
Putative protein sequence of hydra XPF with various features. Bipartite NLS is present from residue 402 to 418 (grey box) with the monopartite NLS (red) contained within it. Residues 576 to 656 make up the ERCC4 domain (green box) with the nuclease motif present from residue 607 to 624 (yellow).

A 2.5 kb cDNA containing the 2451 bp CDS of *XPF* with a 55 bp 5′ UTR and encoding 816 amino acids was isolated from *Hydra vulgaris* Ind-Pune (GenBank accession no. HQ380893). In accordance with the observed (A+T)-rich nature of the hydra genome [27], 69.8% of hydra *XPF* nucleotide sequence is composed of (A+T). Derived *H. vulgaris* Ind-Pune XPF amino acid sequence matched closely with XPF proteins from various animals in BLASTp analysis, indicating a high level of similarity and confirming the identity of the isolated gene as *XPF* (see supporting information Results S1 and Figure S2). Notably, hydra XPF sequence matched better with vertebrate rather than invertebrate homologs (see supporting information Results S1 and Figure S1). The predicted XPF protein contains two types of NLS: a bipartite NLS from residue 402 to 418 and a classic lysine-rich monopartite NLS contained within it (Fig. 1). SMART analysis indicated presence of the conserved ERCC4 domain from residue 576 to 656 of hydra XPF. The ‘GD XnV/IERKX3D’ motif critical for endonuclease activity of the protein [28] is present in hydra XPF from residue 607 to 624 (Fig. 1).

### Homology Modelling Reveals High Structural Similarity between Hydra XPF and Solved Crystal Structures of Various XPF Domains

**Figure 2:**
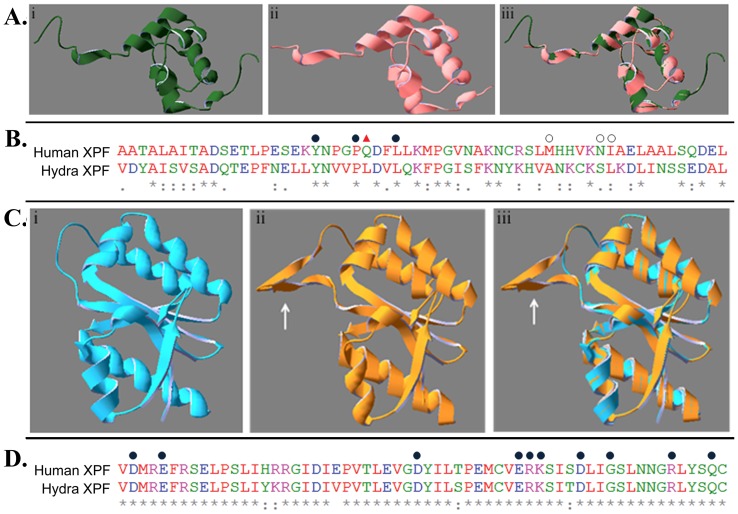
Comparison at structure and sequence levels of hydra XPF regions with corresponding regions from other XPFs. A. Homology modelling for ERCC1-binding domain. i. Structure of B-chain of human XPF-ERCC1 complex (PDB: 1Z00) ii. Predicted structure of ERCC1-binding region of hydra XPF iii. Overlap of 2Ai, 2Aii. **B.** Sequence alignment of ERCC-1 interaction domains from human and hydra XPF. 3 out of 7 residues involved in interaction are conserved (filled circle) while 3 more are replaced by conservative substitutions (open circle). The residue at one position (triangle) is not conserved between the two species. **C.** Homology modelling for the nuclease motif containing ERCC4 domain. i. Structure of *P. furiosus* endonuclease domain (PDB:1J23) ii. Predicted structure of ERCC4 region of hydra XPF iii. Overlap of 2Ci, 2Cii (arrows show extra pair of β-sheets present in hydra XPF) **D.** Alignment of sequence around the nuclease motif of human and hydra XPF. Residues important for catalysis (filled circle) are completely conserved between the two species.

Prediction of hydra XPF structure using automated mode of Swiss Model yielded two models showing similarity with solved crystal structures from the protein data bank (PDB). One constituted the putative ERCC1-binding region (residue 732 to 803) of hydra XPF and used chain B of human XPF-ERCC1 complex [29], (Fig. 2Ai, PDB id: 1Z00) as template. Superimposition of the predicted structure (Fig. 2Aii) and 1Z00 showed a very good fit (Fig. 2Aiii) with root mean square deviation (RMSD) value of 0.08 Å.

The second predicted model covers the ERCC4 domain of hydra XPF (residue 576 to 715) and is based on the structure of *Pyrococcus furiosus* XPF endonuclease domain [30], (Fig. 2Ci, PDB id: 1J23). Superimposition of the generated model (Fig. 2Cii) and 1J23 showed good fit with RMSD value of 0.09 Å (Fig. 2Ciii). An extra pair of beta sheets is present in hydra XPF (Fig. 2Cii, iii, white arrow).

### Hydra XPF Clusters with Early Metazoan and Vertebrate Counterparts in Phylogenetic Analysis

**Figure 3:**
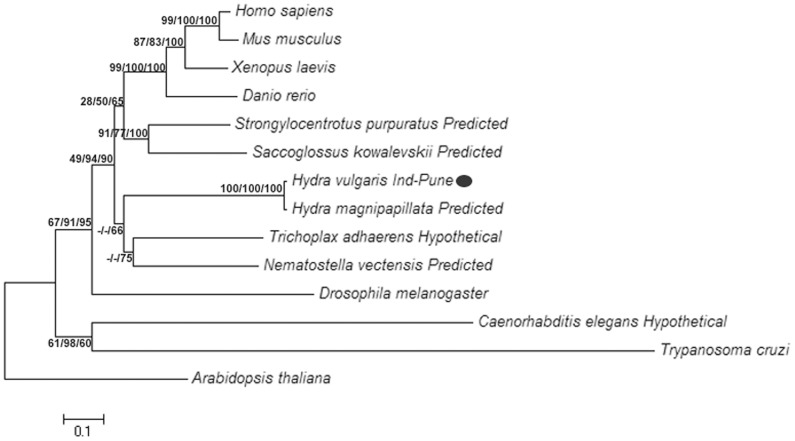
Phylogenetic analysis of XPF protein. A representative NJ tree with branch lengths in units of number of amino acid substitutions per site is shown with bootstrap values for 1000 replicates for ML/MP/NJ trees shown next to branch points. All positions containing gaps and missing data were eliminated for building the trees. Evolutionary distances were computed using the JTT matrix-based method for NJ tree while MP tree was obtained using Close-Neighbour-Interchange algorithm. Initial tree for ML was constructed using BioNJ method and best of Nearest Neighbour Interchange and Subtree Pruning Regrafting was used for tree topology search.

Phylogenetic trees using NJ, MP and ML methods were constructed for XPF protein (Fig. 3). UVH1, the plant homolog of XPF from *Arabidopsis*, was used as an out-group for rooting all trees. In all the trees, vertebrate XPFs grouped together while echinoderm (*Strongylocentrotus*) and hemichordate (*Saccoglossus*) XPFs were found to be closely related to them, forming a cluster of deuterostomes (the taxonomic group of animals where during embryogenesis, blastopore becomes the anus while the second opening forms the mouth). Predicted XPF proteins from *H. magnipapillata* and *H. vulgaris* Ind-Pune grouped with each other in all cases. The protein sequences from *Nematostella*, a cnidarian and *Trichoplax*, a placozoan grouped with hydra XPF sequences forming a cluster of early metazoan XPF proteins. This early metazoan XPF cluster consistently grouped with the deuterostome cluster. XPF proteins from *C. elegans* and the protozoan *Trypanosoma* group together and lie outside the early metazoan-deuterostome cluster along with the *Drosophila* XPF homolog.

### Hydra *XPF* is Strongly Expressed in the Central Region of Body Column and more in Ectoderm than in Endoderm

**Figure 4:**
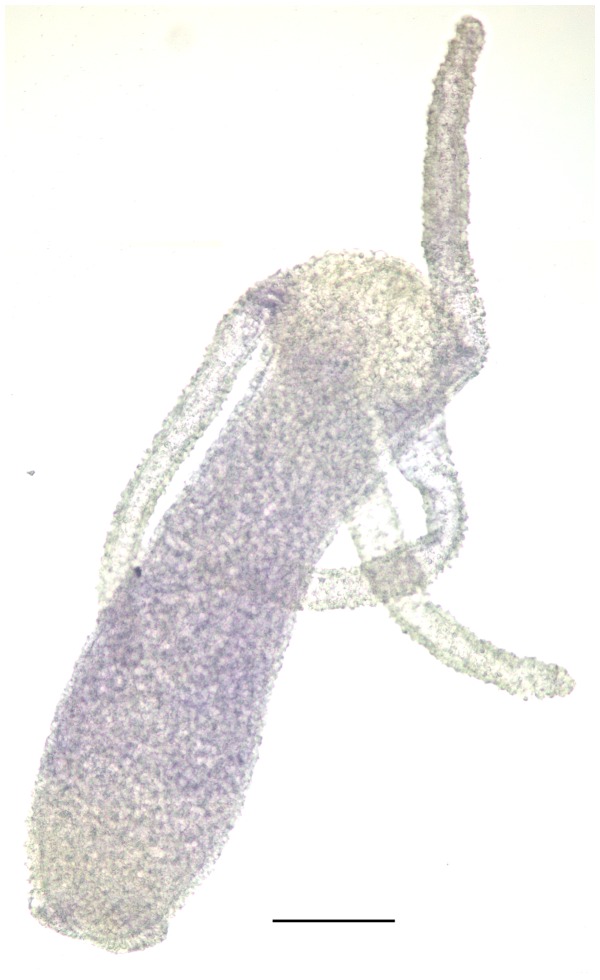
*In situ* hybridization using biotin labelled probes. A representative hydra polyp shows that *XPF* mRNA is present mainly in the central region of the body column but decreases towards extremities. Scale bar = 200 µM.

The expression pattern of *XPF* in adult, non-budding hydra was analysed by whole mount RNA *in situ* hybridization using biotin labelled riboprobe. Expression of *XPF* was strongest in the central region of the body column but diminished towards the extremities, with lower and upper one fourth regions showing hardly any expression (Fig. 4).

**Figure 5:**
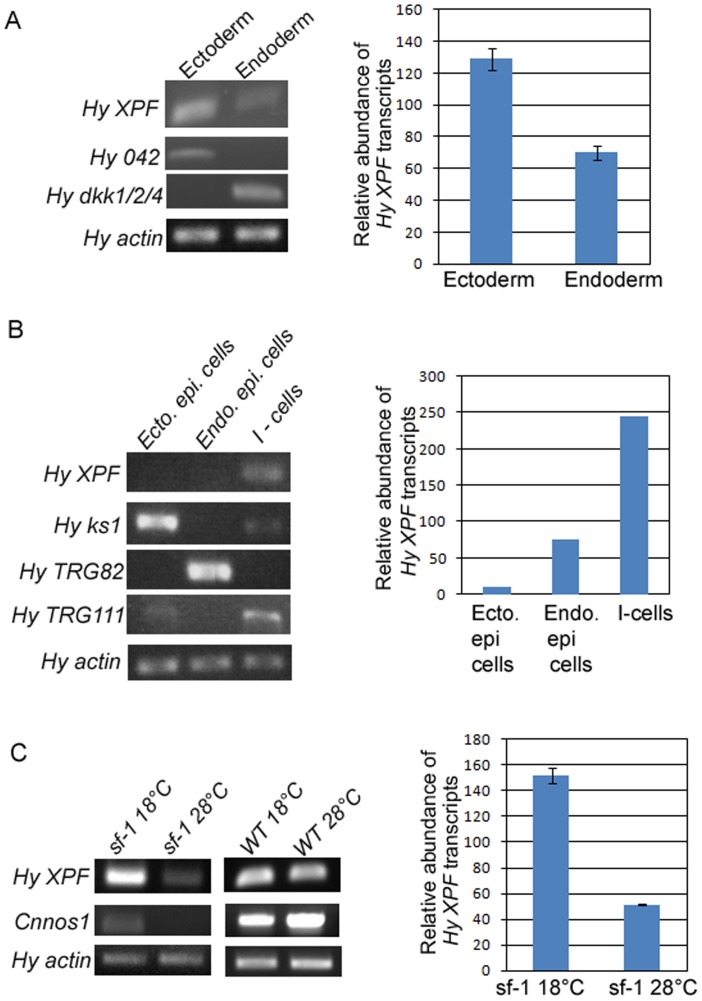
Analysis of expression pattern of hydra *XPF* by semi-quantitative RT-PCR. *Hydra actin* was used as housekeeping control for equilibration in each set. Histogram depicting relative abundance of *XPF* transcripts among the samples is shown adjoining each RT-PCR set. **A.** Level of *XPF* mRNA is nearly twice in ectoderm as compared to endoderm. Ectodermal marker *Hy042* and endodermal marker *HyDkk1/2/4* are expressed predominantly in the respective samples, indicating that the two tissue layers are cleanly separated. **B.** *XPF* expression was compared among three cell types of hydra and was found to be predominant in interstitial cells. Levels of *ks1*, *TRG82* and *TRG111*, markers for ectodermal epithelial cells, endodermal epithelial cells and interstitial cells, respectively, indicate the purity of each cell type fraction. **C.** Prominent expression of *XPF* in interstitial cells was confirmed using sf-1 hydra. *XPF* expression reduced by nearly three-fold upon exposure of sf-1 to 28°C, but remained unchanged in wild-type (WT) hydra. Levels of interstitial stem cell marker *Cnnos1* dropped drastically in sf-1 hydra kept at 28°C indicating loss of interstitial cells but were unaffected in WT hydra, demonstrating that decrease in *XPF* levels is directly correlated with loss of interstitial cells.

To check whether *XPF* is expressed differentially in the two tissue layers of hydra, transcripts were quantified in cleanly separated ectoderm and endoderm by semi-quantitative RT-PCR [25]. Expression of *XPF* in ectoderm of hydra was almost double of that observed in endoderm (Fig. 5A).

### Interstitial Cells of Hydra Prominently Express *XPF*

To screen for variation in expression of *XPF* in three of the main cell types of hydra, namely, ectodermal epithelial cells, endodermal epithelial cells and interstitial cells, semi-quantitative RT-PCR analysis was carried out using cDNA derived from the three cell types. Results indicated that *XPF* is expressed predominantly in the interstitial cells. Expression in interstitial cells was more than three times that of the expression in endodermal epithelial cells while practically no *XPF* expression was detected in ectodermal epithelial cells (Fig. 5B).

To confirm above results we used sf-1, a temperature sensitive variant of *H. magnipapillata* that loses its interstitial cells at non-permissive temperature (28°C) [31], [32]. *XPF* expression was analysed by semi-quantitative RT-PCR in sf-1 and control wild-type *H. magnipapillata* independently exposed to permissive (18°C) and non-permissive temperatures. Levels of interstitial cell marker *Cnnos1* [26] were similar in wild-type *H. magnipapillata* exposed to 18 and 28°C, demonstrating the persistence of interstitial cells after heat treatment. *XPF* expression levels in *H. magnipapillata* samples exposed to the two temperatures also remained comparable, showing that *XPF* expression is not affected by the increased temperature. In sf-1 samples, the loss of interstitial cells in hydra maintained at 28°C was confirmed by cytological examination and greatly reduced expression of *Cnnos1* in comparison with sf-1 animals maintained at 18°C. In these interstitial cell-depleted hydra, expression of *XPF* decreased almost threefold as compared to sf-1 hydra bearing interstitial cells (Fig. 5C). These data show direct correlation between the number of interstitial cells and the level of *XPF* expression and confirm the previous observations.

## Discussion

As no information is presently available about DNA repair in hydra, our study was aimed at characterization of genes involved in this process. We report here the presence in hydra of *XPF*, which encodes a vital constituent without which NER cannot be completed. Its role in a range of processes in addition to NER underlines its importance and warrants investigation of its function and regulation in an evolutionarily ancient organism like hydra. Our findings show that *XPF* is conserved through evolution and the hydra homolog shows the major defining features of the protein, indicating that the gene is present and functional in hydra.

### *In silico* Analysis Shows that Hydra XPF Possesses the Characteristic Features Required for Functioning in the NER Pathway

NLSs mark proteins for import into the nucleus and are a feature of all proteins which act inside it (reviewed in [33]). Correct targeting of XPF protein is crucial for its function as mutations causing mislocalization or inappropriate folding of XPF result in diminished DNA repair [34]. The monopartite and bipartite NLS in hydra XPF (Fig. 1) indicate that it can be targeted to the nucleus suggesting that it is likely to be in the appropriate location to be a part of a functional NER pathway in hydra cells.

ERCC4 domain is a characteristic feature of the XPF/MUS81 family of proteins. It has a very ancient evolutionary history and is present even in XPF homologs from Archaea and earliest eukaryotes [35]. Presence of this domain makes hydra XPF a member of the XPF/MUS81 family of proteins that are involved in repair of DNA lesions.

ERCC4 contains a nuclease sub-domain characterized by the GDXnV/IERKX3D motif which is responsible for endonuclease activity of XPF and hence is its defining feature [28]. In hydra XPF, amino acids 607 to 624 make up this motif. In addition, the mainly acidic residues that correspond to amino acids D676, E679, D704, E714, R715, K716, D720, S724, R729 and Q733 of human XPF are widely conserved and important for interaction with the divalent metal cation (usually Mg^++^) that is required for the catalytic action of XPF [28]. All theses amino acids are present in the hydra XPF at the corresponding locations (Fig. 2D) and along with the nuclease motif, are indicative of the ability of hydra XPF to function as an endonuclease in NER.

The dimerisation of XPF with ERCC1 occurs through the (HhH)_2_ domains present at the C-terminus of both proteins and is critical for their stability, recruitment and positioning at the NER site [29], [35]. The (HhH)_2_ domain consists of two tandem HhH structures separated by another short helix [35]. In human XPF, residues Y833, P837, Q838, L841, M856, N861 and I862 of the (HhH)_2_ motif form a hydrophobic pocket into which a phenylalanine residue of ERCC1 fits [29]. Comparison of *H. vulgaris* Ind-Pune XPF with these amino acids revealed that three of the seven residues are conserved between the two proteins while three other residues are substituted conservatively (Fig. 2B). Thus, hydra XPF may also contain a hydrophobic domain at its C-terminus for interaction with complementary region of a putative hydra ERCC1.

The low RMSD values for superimposition of predicted hydra XPF structures and solved structures from the PDB indicate that hydra XPF is capable of forming the endonuclease and ERCC1-interacting domains, further supporting the possibility of the existence of a functional XPF protein in hydra. The modelling of human and *Drosophila* XPF in Swiss Model also yields structures which are based on *P. furiosus* XPF and are very similar to hydra XPF predicted structure. This shows that domains of XPF proteins from various organisms form structures that are very similar to each other, indicating the early evolution of XPF protein and its conservation across phyla and even across kingdoms.

### Basal Metazoan XPF Proteins Cluster with Vertebrate Homologs in Phylogenetic Analysis

*XPF* gene is evolutionarily ancient. It is present in some archaea and across eukaryotes in fungi, plants and animals [35]. In Cnidaria, the most basal members of the eumetazoa, sequences showing similarity with *XPF* were found in hydra and *Nematostella*. We analysed the phylogenetic relationship of XPF proteins from various animals with an emphasis on early metazoan and invertebrate phyla. The analysis further substantiates the initial observation that hydra XPF protein is closely related to vertebrate XPFs. The cluster of early metazoan XPFs groups with the cluster formed by deuterostome XPFs, indicating their relatedness at the sequence level. Consistently high bootstrap values at the node between (early metazoan-deuterostome) cluster and the remaining sequences demonstrate the reliability of this grouping. It has been previously observed that despite diverging very early in evolution cnidarian gene sequences are often more similar to vertebrate sequences than to counterparts in model invertebrates, indicating their high level of complexity [27], [36]. The NER gene *XP group B* from the sponge *Geodia* has also been observed to be very similar to its human homolog [13]. In our analysis, the extended branch lengths for *Drosophila*, *Caenorhabditis* and *Trypanosoma* XPFs indicate that the sequences from these animals are distant from the other XPFs analysed, possibly due to rapid rates of genome change in some of these model invertebrates that resulted in the divergence of their genomes away from ancestral metazoan sequences [27], [36]. Present findings corroborate previous observations and support the view that vestiges of genome organization from the ancestor of eumetazoa persists in hydra [27], [36].

In view of the high divergence in genomes of model invertebrates like *Drosophila* and *Caenorhabditis*, the observed similarity of hydra XPF to vertebrate homologs underlines the significance of hydra as an interesting alternative model in study of metazoan evolution.

### Prominent Expression of *XPF* in Interstitial Cells has Interesting Implications for Germ Line Protection and Somatic Cell Turn Over in Hydra

Ectoderm, being the outer layer of the body, is more exposed to potentially harmful DNA damaging agents like UV radiation or other mutagens in the surroundings. Expressing higher amounts of DNA repair genes like *XPF* in the ectoderm could be one way to overcome the damage caused by such agents. Immunostaining with an interstitial cell specific antibody and analysis of the distribution of various cell types in hydra has shown that interstitial cells are present mainly in the body column and absent from the extremities [3], [37]. The *in situ* hybridization pattern of hydra *XPF* thus overlaps the location of interstitial cells. Predominant expression of *XPF* in interstitial cells as observed in subsequent experiments may account for the high *XPF* levels detected in the ectoderm and in the central region of body column as this is where interstitial cells reside [3].

**Figure 6:**
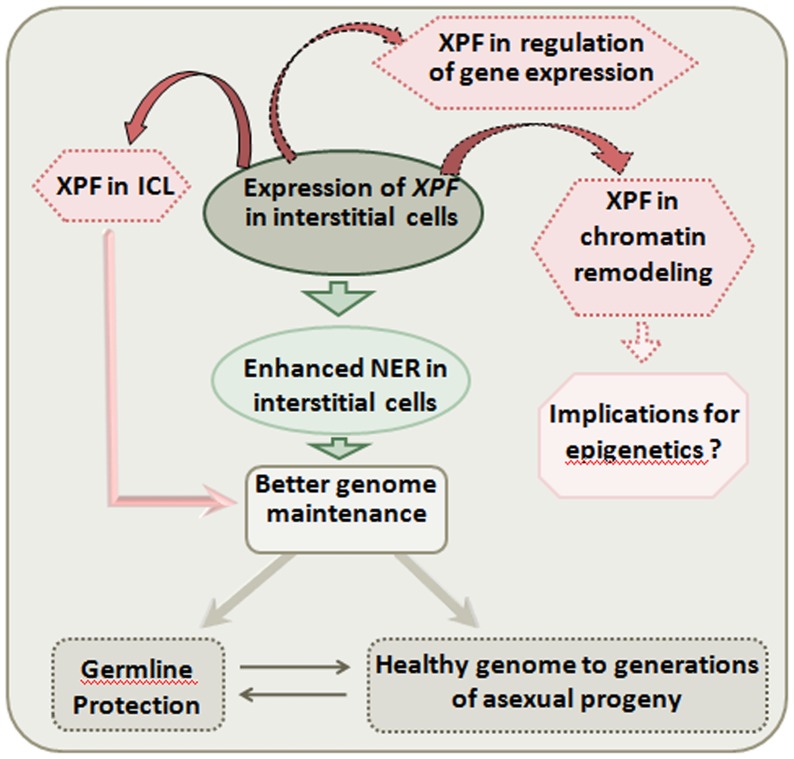
Possible implications of high *XPF* expression in interstitial cells. Primarily, *XPF* expression indicates that NER may be enhanced in interstitial cells leading to better genome maintenance, which in turn can result in protection of germline and asexual progeny with a healthier genome. High expression of *XPF* in interstitial cells may also have effects on processes other than NER. Its role in interstrand cross-link repair may affect genome maintenance while other putative roles could have implications for chromatin remodelling and regulation of expression of certain genes.

Interstitial cells constitute the multipotent stem cell lineage of hydra that gives rise to a wide range of cell types including nematocytes, gland cells, neurons and gametes. Interstitial cells and their derivatives constitute nearly 80% of all cells present in a polyp (reviewed in [3]). The high expression of a DNA repair gene like *XPF* in these cells could have several implications (Fig. 6).

Generally germ cells of an animal are specially protected against genomic damage [38]. Genes related to DNA repair and its regulation show enhanced expression during gametogenesis in mammals [39], while in *C. elegans,* germ cells are set apart very early in development [40]. In hydra, germ cells are not segregated. Interstitial cells constitute the germ line and produce gametes [3]. Elevated levels of a repair gene like *XPF* in these cells, appears to be a mechanism to guard the genome that is passed on to the next generation. Hydra interstitial cells express high levels of *FoxO* [41], a member of the gene family known to be involved in stress response and protective mechanisms like promotion of longevity during dietary restriction in *C. elegans* [42], protection of quiescent cells from oxidative stress [43] and even modulation of cellular stress-resistance through induction of DNA repair [44]. High expression of *FoxO* in interstitial cells of hydra supports the notion that damage response and protective genes like *XPF* are active in these cells.

Stem cells in the body column of hydra divide continuously (reviewed in [3], [45]) and are hence responsible for the constant renewal of body tissues and bud formation throughout the potentially unlimited life of hydra. Despite the continuous cell division, reports of incidence of cancer in hydra have been exceedingly rare [1]. Out of the three stem cell types in hydra, interstitial cells have the fastest rate of cycling [3]. A hydra polyp constantly renews its tissues with new cells and reproduces asexually throughout its potentially unending life [4]. The interstitial stem cells thus undergo a large number of cell divisions. It is well known that deficient DNA repair exponentially increases the risk of cancer [46]. Robust genome protection is therefore essential for continued survival and reproduction. High expression of *XPF* along with other repair genes could be a means to ensure that an intact, error-free genome is maintained in the tissues of the animal and passed on to its asexual and sexual progeny.

Recent reports suggest that NER factors including XPF may have a role in histone modification, demethylation and removal of 5-methyl cytosine [17], [18]. In this light, high expression of *XPF* in the multipotent and fast cycling interstitial stem cells of hydra is of added interest and merits further enquiry into functions and regulation of *XPF* in this potentially immortal animal.

In conclusion, this study demonstrates the presence of *XPF* in hydra for the first time and shows that the gene is conserved at sequence, domain and structural levels. Given the evolutionary distance between hydra and vertebrates, the observed similarity of hydra XPF with its vertebrate rather than invertebrate homologs is especially noteworthy. The finding that *XPF* is expressed at a high level in the interstitial stem cells that also constitute the germ line of the non-senescent hydra is significant. The data indicate that *XPF* has an important function in hydra and provide a framework for future lines of investigation. Other studies from our laboratory have shown for the first time that other genes of NER: *XPA, XPB, XPC, XPD, XPE, CSA* and *CSB* are also present and expressed in hydra and partial coding sequences of these genes have been submitted by us to NCBI GenBank database (accession numbers: JN411719, JN411718, JN411717, JN411716, JN4115, JQ822227 and JQ822228 respectively). The partial sequence of *XPG* has also been isolated and is currently under submission to GenBank. We have cloned and partially characterized the complete CDS of hydra *XPA* (Barve, A., Ghaskadbi, S. and Ghaskadbi, S., manuscript under preparation). Taken together, these observations strongly suggest that the NER pathway is functional in hydra. Analysis of the other DNA repair genes and their regulation will enable us to draw broader conclusions and build testable hypotheses regarding the role of DNA repair in the physiology of the remarkable hydra.

## Supporting Information

Figure S1 **Multiple sequence alignment of hydra, human, zebrafish,** ***Drosophila*** **and** ***Caenorhabditis*** **XPF amino acid sequences.** Hydra XPF shows high level of similarity to its homologs from other animals and especially to vertebrate XPFs.
(TIF)

Figure S2 **Multiple sequence alignment of hydra XPF, human XPF and human MUS81 amino acid sequences.** Hydra XPF amino acid sequence is highly similar to human XPF and shows almost no similarity with human MUS81, clearly establishing its identity as a XPF protein.
(TIF)

Results S1 **Analysis of sequence isolated from hydra.** Comparison of hydra sequence with other related sequences from the database demonstrates its identity as XPF and shows that it is more similar to vertebrate, than invertebrate XPFs.
(DOC)
