# Supplementary figures and images for: Conservation of the Nucleotide Excision Repair Pathway: Characterization of Hydra Xeroderma Pigmentosum Group F Homolog
Source: PLoS One. 2013 Apr 8;8(4):e61062. doi: 10.1371/journal.pone.0061062 (PMC3620063; doi:10.1371/journal.pone.0061062)

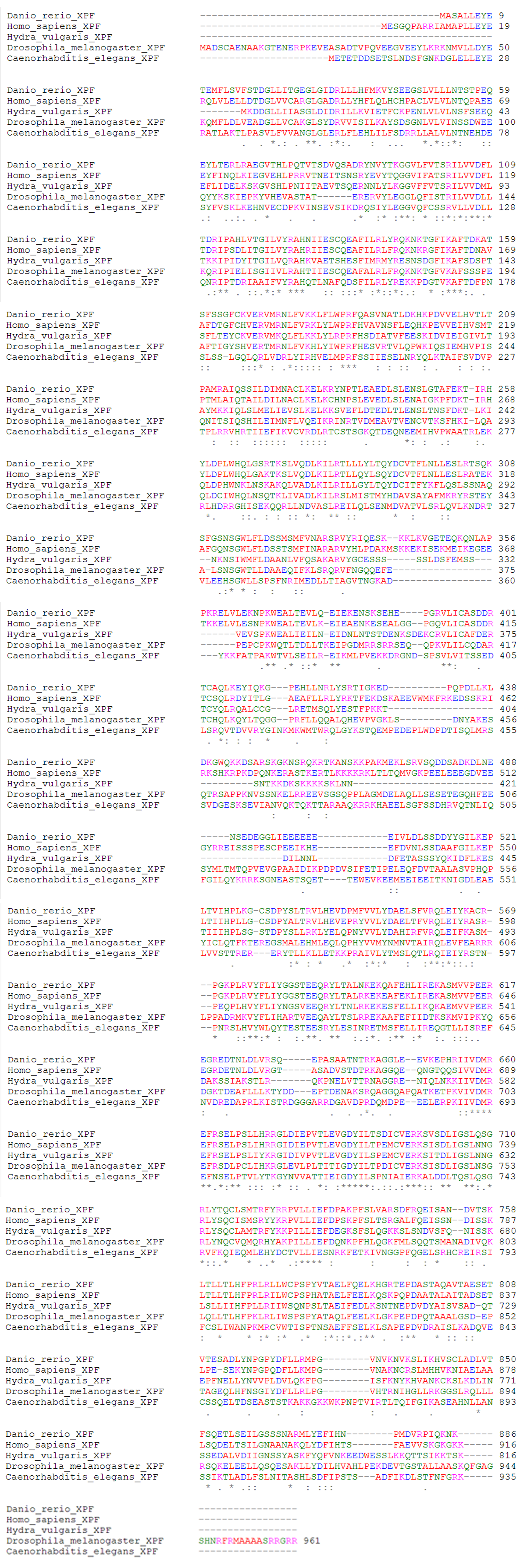

Supplement: Figure S1 — Multiple sequence alignment of hydra, human, zebrafish, Drosophila and Caenorhabditis XPF amino acid sequences. Hydra XPF shows high level of similarity to its homologs from other animals and especially to vertebrate XPFs. (TIF) [file pone.0061062.s001.tif]

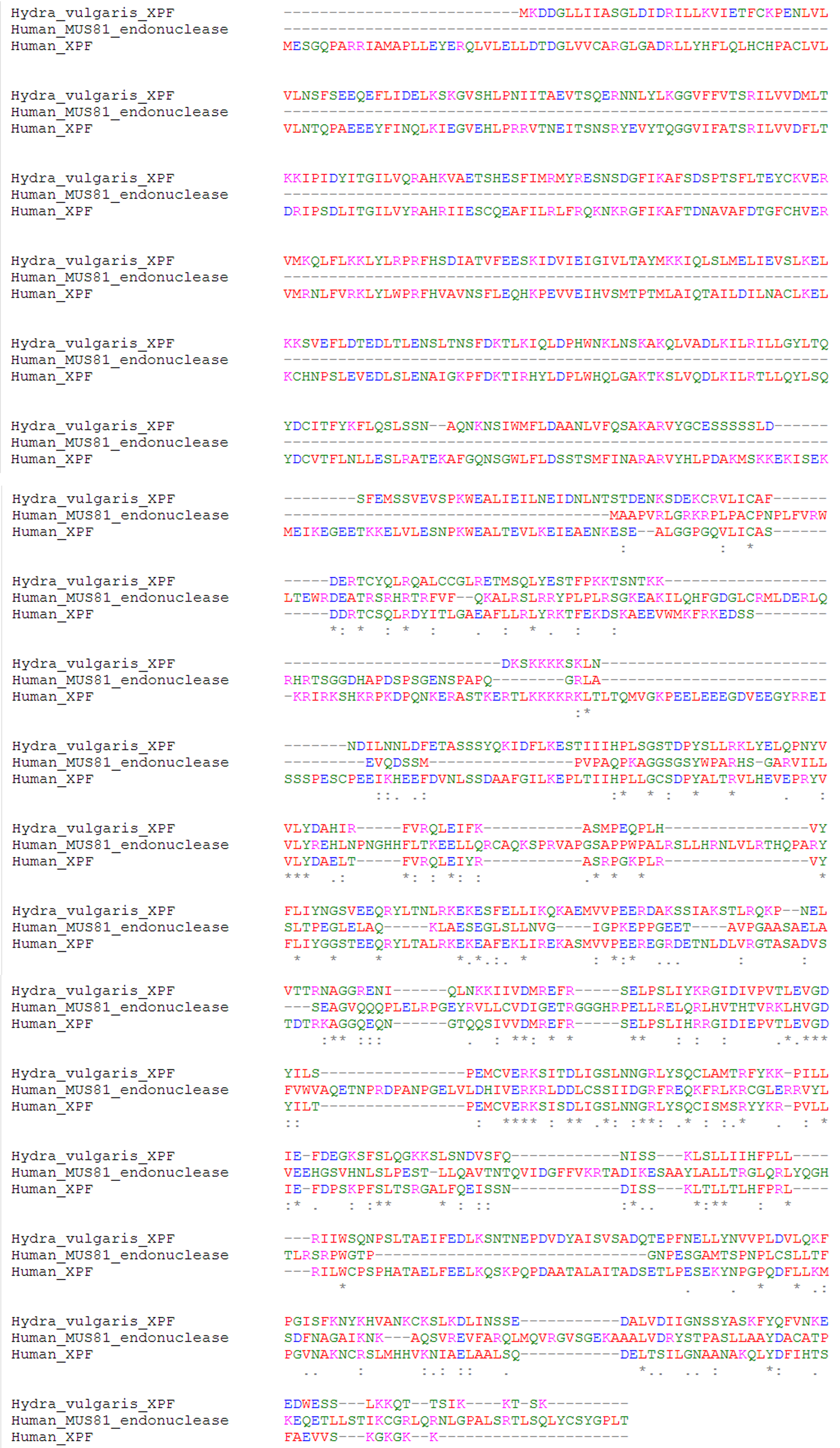

Supplement: Figure S2 — Multiple sequence alignment of hydra XPF, human XPF and human MUS81 amino acid sequences. Hydra XPF amino acid sequence is highly similar to human XPF and shows almost no similarity with human MUS81, clearly establishing its identity as a XPF protein. (TIF) [file pone.0061062.s002.tif]
