# Supplementary material for: Conservation of the Nucleotide Excision Repair Pathway: Characterization of Hydra Xeroderma Pigmentosum Group F Homolog
Source: PLoS One. 2013 Apr 8;8(4):e61062. doi: 10.1371/journal.pone.0061062 (PMC3620063; doi:10.1371/journal.pone.0061062)
Supplement: Results S1 — Analysis of sequence isolated from hydra. Comparison of hydra sequence with other related sequences from the database demonstrates its identity as XPF and shows that it is more similar to vertebrate, than invertebrate XPFs. (DOC) [file pone.0061062.s003.doc]

**Comparison of hydra sequence with other related sequences from the database to demonstrate its identity as XPF and show its close similarity to vertebrate XPFs**

**BLASTp Analysis**

When the putative protein sequence of hydra XPF was used as a query to carry out BLASTp analysis at the NCBI server, it showed notable similarity with other XPF proteins. Four out of top five hits of hydra XPF BLASTp analysis are vertebrate XPFs, while the fifth one is the predicted sequence of XPF from another species of hydra (See Table S1) and majority of the top 100 hits were vertebrates.

Table S1. Scores for top five hits of BLASTp analysis of hydra XPF

| **Description** | **Accession no.** | **Organism** | **Scores (Bits)** | **E value** |
| --- | --- | --- | --- | --- |
| PRED.: similar to DNA repair endonuclease XPF | XP_002161035.1 | *Hydra magnipapillata* | 1510 | 0 |
| PRED.: similar to DNA repair endonuclease XPF | XP_003417763.1 | *Loxodonta africana* | 761 | 0 |
| DNA repair endonuclease XPF | NP_956079.1 | *Danio rerio* | 760 | 0 |
| DNA repair endonuclease XPF | ACO52467.1 | *Liza aurata* | 758 | 0 |
| DNA repair endonuclease XPF | Q9QYM7.3 | *Cricetulus griseus* | 759 | 0 |

**Sequence alignment**

Multiple sequence alignment of XPF protein sequences from hydra, zebrafish, human, *Drosophila* and *Caenorhabditis* was carried out. This shows that hydra XPF is very similar to human and zebrafish XPF (Figure S1).

One-on-one amino acid sequence alignments of hydra XPF sequence with two invertebrate (*Caenorhabditis* and *Drosophila*) and two vertebrate (human and zebrafish) XPFs were carried out. All parameters like total score, maximum score and maximum identity clearly demonstrate that hydra XPF is more similar to vertebrate homologs that to those from *Drosophila* or *Caenorhabditis* (see Table S2).

Table S2. Scores for alignment of hydra XPF with zebrafish, human, *Drosophila* and *Caenorhabditis* XPFs

| **Description** | **Acc. No.** | **Max. Score** | **Total score** | **Query cover** | **E value** | **Max. Identity** |
| --- | --- | --- | --- | --- | --- | --- |
| Zebrafish | NP_956079 | 760 | 760 | 97% | 0.0 | 46% |
| Human | NP_005227.1 | 418 | 826 | 95% | 2e-135 | 55% |
| *Drosophila* | NP_525068 | 497 | 497 | 97% | 4e-165 | 35% |
| *Caenorhabditis* | NP_496498 | 189 | 345 | 81% | 8e-54 | 37% |

**Comparison of hydra sequence with human XPF and MUS81**

In order to eliminate the possibility that the sequence isolated from hydra is more closely related to MUS81 (a paralog of XPF) than to XPF, multiple sequence alignment and one-to-one comparison of hydra sequence with human XPF (NP_005227.1) and human MUS81 (AAL28065.1) was carried out. This clearly showed that hydra XPF is much more similar to human XPF than to MUS81 (Figure S2). Though MUS81 bears the ERCC4 domain, it is substantially shorter than XPF proteins. Hydra XPF is comparable in length to human XPF. One to one sequence alignment proves that on all parameters like total score, maximum score and maximum identity, the hydra sequence shows very high level of similarity with human XPF, but extremely low scores when compared with human MUS81 protein. This demonstrates clearly that the sequence from hydra is XPF and not MUS81 (see Table S3).

Table S3. Scores for alignment of hydra XPF with human XPF and human MUS81

| **Description** | **Acc. No.** | **Max. Score** | **Total score** | **Query cover** | **E value** | **Max. Identity** |
| --- | --- | --- | --- | --- | --- | --- |
| Human XPF | NP_005227.1 | 418 | 826 | 95% | 2e-135 | 55% |
| Human MUS81 | AAL28065.1 | 22.7 | 45 | 7% | 0.056 | 44% |
